# mHealth Support in Cardiac Care Pathways for Patient Self-Management During Transitions From Hospital to Rehabilitation: Exploratory Field Study

**DOI:** 10.2196/76089

**Published:** 2025-08-27

**Authors:** Isabel Höppchen, Stefan Tino Kulnik, Alexander Meschtscherjakov, Josef Niebauer, Bernhard Reich, Jan David Smeddinck, Daniela Wurhofer

**Affiliations:** 1 Ludwig Boltzmann Institute for Digital Health and Prevention, Lindhofstr. 22, Salzburg, 5020, Austria, 43 572558270; 2 University of Salzburg, Salzburg, Austria; 3 University Institute of Sports Medicine, Prevention and Rehabilitation, Paracelsus Medical University, Salzburg, Austria; 4 REHA Zentrum Salzburg, Salzburg, Austria

**Keywords:** cardiac rehabilitation, mHealth, digital health, empowerment, patient transitions, telemedicine, transitional care, usability, mobile health

## Abstract

**Background:**

Cardiac rehabilitation (CR) is essential for recovery from cardiovascular disease. However, patients often encounter challenges in navigating the transition from acute hospital care to CR. Mobile health (mHealth) technologies may support this critical phase; however, evidence regarding their clinical practice remains limited. The HERO app (developed by REDOX GmbH) was developed to address the needs of patients with cardiovascular disease for orientation, emotional support, and motivation during this transition.

**Objective:**

This study aims (1) to explore how mHealth technologies tailored for patients with cardiovascular disease can support their needs regarding orientation, emotional balance, and motivation during the transition from the acute hospital to CR and (2) to evaluate the user experience and acceptance of the HERO app as targeted pathway support.

**Methods:**

A mixed methods study was conducted with patients with cardiovascular disease using study diaries, questionnaires, and semistructured interviews. Participants were purposively recruited in acute hospitals and rehabilitation settings. Quantitative data were analyzed descriptively, and qualitative data were analyzed using content analysis after Mayring.

**Results:**

Eight participants used the app for an average of 14 (range 4-23) days. The app was perceived as a helpful short-term resource. It supported patients in understanding their condition, planning for CR, and regaining motivation. Participants highlighted the value of combining objective information with peer experiences. Suggestions for improvement included more personalized self-management guidance and a precise onboarding process to increase accessibility and usability.

**Conclusions:**

Based on the findings, we propose 4 pillars of mHealth support for cardiac care transitions, including timely access, actionable guidance, peer support, and short-term usability. These pillars could inform the design of patient-centered mHealth tools for care transitions.

## Introduction

Cardiac rehabilitation (CR) is highly recommended by international guidelines as a class 1 indication for many patients with cardiovascular disease, for example, after an acute myocardial infarction [1]. However, participation rates are low, with approximately only 34% of eligible patients globally taking up CR [2]. Missing out on rehabilitation can have profound implications on patients’ long-term morbidity and mortality [3]. Early uptake of CR after hospitalization is beneficial to mitigate potential uncertainty about the cardiac condition, for example, by learning coping strategies [4,5]. Nevertheless, transitions from the acute hospital to rehabilitation often include multiple steps and can be complex and challenging for patients [6,7].

In a previous study, we found that after discharge from the acute hospital, patients experience primarily three needs: (1) a "need for emotional balance" to reflect on the cardiac event and the time in the hospital; (2) a "need for orientation" to get to know and consider follow-up care options, such as CR; and (3) a "need for motivation" for a healthy lifestyle, including the participation in CR [8]. Adequate and timely information can help address these needs, but clinical practice to date fails to address these needs and implement effective solutions [9,10].

Information provision should ideally commence early after an acute cardiac event to increase patients’ confidence in managing their condition and improve the likelihood of CR uptake [10]. However, access to reliable information sources is often limited to the patients’ hospital stay, as health care professionals are their primary information sources [11,12]. Previous research indicated a mismatch between the amount of information health care professionals provide and patients’ ability to capture it [13]. Information provision during hospitalization is also often provided verbally and low-technology methods, constraining the possibility of rereading and processing beyond patients’ hospital stay [13].

After discharge, patients often struggle with self-assessing their physical capacity and adopting healthy behaviors, such as dietary changes, exercising, and smoking cessation [14]. During this critical step in the patient pathway, patients need to know how to self-manage their condition. Peer support, goal setting, and access to health services can be adequate resources for patient support [13]. Mobile health (mHealth) technologies have the potential to provide access to reliable information beyond patients’ acute hospital stay and, therefore, widen the time window for processing information [13]. They could offer tailored information according to the rehabilitation progress, adapted to patients’ health literacy and individual care pathways across health care sectors [8,11,15].

Several studies found evidence that mHealth could increase patients’ knowledge and, thereby, empower them to actively participate in their care process and decision-making [16-19]. Vardoulakis et al [19] provide an example of how a smartphone app can affect patients’ knowledge by providing layman-friendly health information. The interaction with the app increased patients’ knowledge about their care plan and allowed them to actively participate during daily ward rounds, for example, by asking specific questions [19]. Also, the patients appreciated that the app removed dependence on health care professionals to provide information. Being aware of the possibility to reread details had a calming effect on them [19].

Despite the potential for effective mHealth support, there is a lack of evidence about digital support for intersectoral, multistep patient pathways. Related studies often focus exclusively on specific health care touchpoints, such as outpatient care or long-term lifestyle adjustments [20-22]. Less is known about the targeted support needed during phases when patients find themselves between 2 health care touchpoints. In light of this, we developed the “Dein Weg zur Reha” (“Your pathway to rehabilitation”) smartphone app (HERO app) to support the evolving needs of patients with cardiovascular disease along their care pathway [8].

This exploratory field study investigated how an mHealth technology, such as the HERO app, could address the informational and emotional needs of patients during critical transitions from the acute hospital to CR. The objectives of this study were (1) to explore how mHealth technologies tailored for patients with cardiovascular disease can support their needs regarding orientation, emotional balance, and motivation during the transition from the acute hospital to CR, and (2) to evaluate the user experience and acceptance of the HERO app.

## Methods

### Overview

This exploratory field study investigated how the HERO app could support patients with cardiovascular disease during their transition from the acute hospital to CR. To capture user experiences from a naturalistic use setting and to assess the app’s sociotechnical context [23,24], we integrated the app into the patient pathway after hospitalization.

### The HERO App for Tailored Patient Support

**Figure 1.**
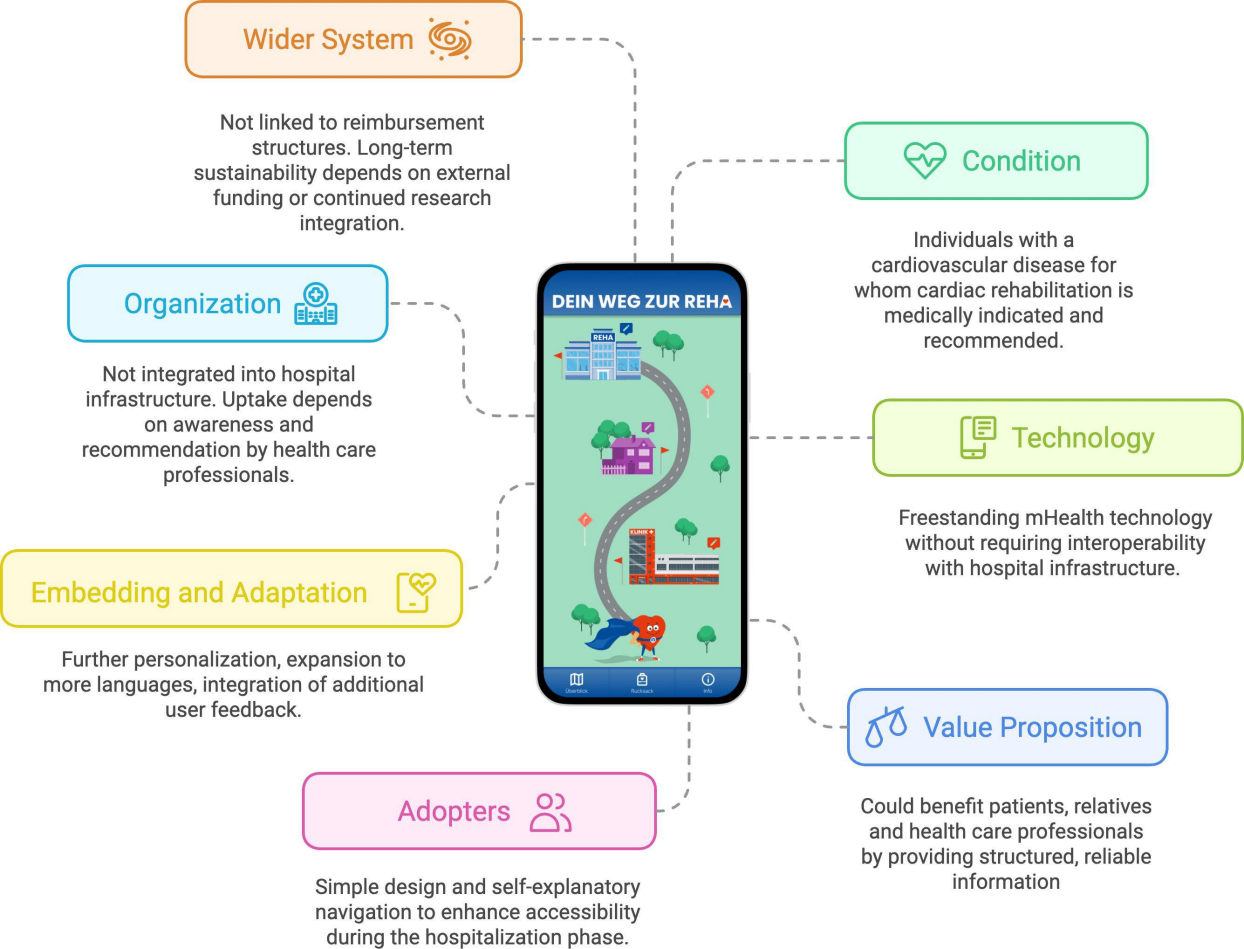
The HERO app and its use context. mHealth: mobile health.

This study builds on previous research, including a contextual inquiry [6] and an in-depth understanding of the needs of patients with cardiovascular disease along care pathways [8]. The results informed the development of the HERO app, which aims to address patients’ needs along their care pathway by providing necessary and reliable information. Figure 1 illustrates the HERO app interface and its application context within the patient pathway, mapped along the domains of the NASSS (Nonadoption, Abandonment, Scale-Up, Spread and Sustainability) framework, including technology, users, organization, and wider system. Multimedia Appendix 1 presents a video walkthrough of the app.

In the following, we describe the HERO app according to the domains of the NASSS framework, developed by Greenhalgh et al [25]. The NASSS could be used to understand and evaluate the challenges of implementing technologies in health care systems.

The app was developed using React Native and is provided as an Android Package Kit (mobile app). It requires a minimum operating system version of Android 5.0. Users must have an Android smartphone and an active internet connection to access the app and linked content.

The target users of the HERO app are individuals with cardiovascular disease for whom CR is medically indicated and recommended. Informal caregivers and relatives could also benefit from the information provided in the app. In addition, health care professionals may find the app valuable as it offers a time-saving method for delivering information and educational content during daily ward rounds. The app is designed to provide self-explanatory content and intuitive navigation, ensuring that health care professionals do not need to spend time introducing patients to its use.

The HERO app is a standalone mHealth technology and can be used offline and without entering personal data to avoid potential privacy concerns. The decision to design the app without requiring interoperability with other technologies on a patient ward was intentional to be easily downloaded onto patients’ smartphones without complex integration into hospital infrastructure.

The home screen presents a visualization of a patient pathway from hospital to CR, including the 3 steps “Hospital,” “Home,” and “Rehabilitation” [26]. Within these 3 steps, users find evidence-based information about CR and experiential knowledge in videos with testimonials from 2 former CR participants [27,28]. The app offers most content bilingually in German and English to accommodate users with different language preferences within the Austrian health care context. The content changes from step to step to meet the temporal dimension of patients’ information needs and processing [8,11]. We also included a “backpack” function, in which users can save preferred information and take notes, taking these with them from one step to the next.

### Recruitment Strategy and Inclusion Criteria

We recruited patients with cardiovascular disease between August 2024 and March 2025. Following a purposive sampling strategy, participants were recruited by 2 gatekeepers in hospitals located in different federal states in Austria. The gatekeepers were personal contacts of the project lead and senior physicians in cardiology wards. The gatekeepers screened patients against the inclusion criteria and provided them with a flyer about the study, contact details of the project lead, and a web link to sign up for the study. Patients could also give their telephone numbers and agree to be contacted by the project lead for recruitment purposes. After the patients gave written consent to participate in the study, they were provided with a study diary and a link to download the app on their smartphones.

Inclusion criteria were hospitalization due to a recent acute cardiac event, a newly diagnosed cardiovascular disease with an indication for attending or currently attending CR in Austria, using a smartphone with an Android operating system, and willingness to install the HERO app on that phone. Exclusion criteria were using a smartphone with an iOS operating system, age less than 18 years, inability to give informed consent, or inability to participate due to the health condition. The recruitment proved to be exceptionally challenging in the hospital setting. A notable barrier was the limitation that the HERO app was only available for Android. Many eligible patients used iPhones (Apple Inc) and declined the offer to borrow an Android phone during the study period. Further reasons included participation in other studies and limited physical or mental energy to participate in research. In some cases, patients could not be reached again after initially expressing interest. Therefore, we decided to broaden the inclusion criteria to also include individuals who were currently participating in a CR program in an outpatient rehabilitation center.

### Quantitative and Qualitative Measures

For the app-testing phase, participants received a paper-based study diary. It included onboarding activities to get to know the features of the app, such as “Save a relevant information card in the backpack” or “Go to the ‘Hospital’ and watch a video.” Participants were asked to complete the AttrakDiff questionnaire for user experience [29], the Unified Theory of Acceptance and Use of Technology 2 (UTAUT2) questionnaire for user acceptance [30], and a questionnaire about the frequency of app use. In addition, open questions and space for notes were included to encourage reflection on the overall user experience and interaction with the HERO app. After the app-testing phase, we conducted semistructured interviews with the participants to capture their experiences with the app and to explore to what extent the app had an impact on their pathway to CR. For participants who were recruited during their CR program, interview questions were framed to encourage hypothetical reflection on how the HERO app might have supported them during the earlier transition phase. The interview guide can be found in Multimedia Appendix 2. All interviews were conducted by the project lead and were audiotaped. The interview language was German, and illustrative quotes for the “Results” section of this paper were translated into English.

### Dataset and Analysis

The quantitative dataset consisted of the AttrakDiff, UTAUT2, and frequency of use questionnaire. This data were analyzed descriptively using Excel software (Microsoft). The qualitative dataset consisted of participants’ diary entries and 193 minutes of interview recordings (mean 24; range 13-40 minutes). Audio recordings were transcribed verbatim by the project lead and supported by f4x software (Dr. Dresing & Pehl GmbH). To protect participants’ confidentiality, identifying information was removed and data were pseudonymized prior to analysis. The transcripts were analyzed following qualitative content analysis after Mayring [31], a structured approach to systematically categorize qualitative data. To begin, we defined a coding guide with overarching categories (Multimedia Appendix 3). These categories were derived from our research questions, our previous study on patient needs [8], and the perception of user experience provided by Hasselzahl et al [32]. Categories were then applied to the data and further refined inductively into subcategories to achieve a fine-grained analysis. In line with Mayring [31], we defined the “coding unit” [31] as multiple words sharing a common meaning to ensure a consistent coding approach. The “context unit” [31], which provided interpretive background, included the entire interviews, diary entries, and demographic questionnaires. Finally, we considered the “analysis units” [31] to be the diary entries and the questionnaire responses. The paraphrased transcripts were then coded with the category system by the project lead, supported by MAXQDA 2022 software (VERBI Software). The category system and interpretations were discussed within the research team to ensure consistency and reliability.

### Ethical Considerations

The study was reviewed by the research ethics committee of the Ludwig Boltzmann Gesellschaft in Austria and received a favorable opinion (reference 014_2024). All patients gave written consent to participate in the study. After study completion, they received a compensation of €30 (US $35.17).

## Results

### Overview

In the following, we present participant characteristics (Table 1) and overall feedback on the HERO app’s usability, aesthetics, and use context. We provide insights into the five key themes that emerged from the data: (1) Considering and preparing for cardiac rehabilitation, (2) enhancing motivation to return to daily activities, (3) supporting sense-making of the cardiac event, (4) ensuring timely and location-independent access to the app, and (5) improving self-management guidance and addressing information gaps.

**Table 1.** Participant characteristics and interview settings.

ID	Sex	Age (years)	Profession	Cardiovascular diagnosis	Year of diagnosis	Participated in CR^a^	Interview setting
P01	Male	54	Insurance employee	Myocardial infarction, status post stent implantation	2024	Yes (first time)	Face-to-face interview at his home before he started CR
P02	Male	59	Company technician	Myocardial infarction, status post stent implantation	2024	Yes (first time)	Telephone interview before he started CR
P03	Male	74	Artist, retired teacher	Myocardial infarction, status post stent implantation	2019	No	Telephone interview after he decided not to participate in CR
P04	Female	54	Kindergarten teacher	Acute coronary symptom (NSTEMI)^b^	2024	Yes (first time)	Telephone interview during CR
P05	Female	32	Teacher	Peripartum cardiomyopathy, functional mitral insufficiency	2024	Yes (first time)	Face-to-face interview during CR
P06	Male	66	Retired coach for corporate development	Myocardial infarction, status post stent implantation	2012	Yes (second time)	Face-to-face interview during CR
P07	Female	77	Retired medical technical assistant	Coronary heart disease	2012	Yes (fourth time)	Face-to-face interview during CR
P08	Male	57	Logistics manager	Coronary heart disease	2024	Yes (first time)	Telephone interview during CR

a CR: cardiac rehabilitation.

b NSTEMI: non-ST-elevation myocardial infarction

### Participant Characteristics

The study population consisted of 8 participants who were recruited in 3 different federal states in Austria. The mean age was 59.1 (range 32-77) years, and the majority were male (5 self-identifying males and 3 self-identifying females). A total of 4 participants were recruited during hospitalization and 4 during their CR program. While 2 participants were referred by the hospital staff, 4 were referred by their general practitioner or by their internist, and 1 initiated and administered the referral himself. For CR attendants who were hospitalized, the average time between discharge and the start of CR was 69 (range 24-165) days. Overall, 5 participants attended CR for the first time, 2 had attended CR previously, and 1 declined CR participation.

### Overall Feedback on Usability, Aesthetics, and Use Context

The participants used the HERO app for 14 (range 4-23) days on average. Users’ UTAUT2 and AttrakDiff ratings are presented in Table 2. The users stated that the app use slightly became a habit (median 4, IQR 1-5).

**Table 2.** Unified Theory of Acceptance and Use of Technology 2 (UTAUT2) and AttrakDiff ratings from study participants (n=8).

Questionnaire (score range) and dimension			Median (IQR)
UTAUT2 (range 1 to 7, higher score indicating better user acceptance)			
Habituation			4.0 (1-5)
Perceived usefulness			4.5 (2-6)
Acquired knowledge			6.0 (3-7)
Intention to use			2.5 (1-6)
Experienced enjoyment			5.0 (4-6)
AttrakDiff (range –3 to 3, higher score indicating better user experience)			
Pragmatic quality			2.0 (–3 to 3)
Hedonic quality–stimulation			0.5 (–3 to 3)
Hedonic quality–identity			1.0 (–1 to 3)
Attractiveness			1 (–1 to 3)

The low intention to use the app in the future (median 2.5, IQR 1-6) contrasts with generally positive ratings for usefulness and enjoyment (Table 2). This arguably reflects the app’s short-term intended use rather than dissatisfaction. Some individuals described the app as a “tool” (P06) used mainly between discharge and the start of CR, after which it could be set aside. For example, participant “P04” indicated that she consulted the app multiple times at home but discontinued use once CR started, as she no longer had the time or a need for it.

Most participants reported having the necessary knowledge to use the app (median 6, IQR 3-6). Only participant “P03” expressed skepticism about digital technologies in general and a lack of familiarity with installation and navigation. The pragmatic quality ratings varied (median 2, IQR –3 to 3), indicating different perceptions of user-friendliness and functionality. Most participants emphasized the app’s straightforward design and ease of use, noting that its user guidance was largely self-explanatory. This would make the app “easy to handle even for patients in a hospital bed*”* (P05). Participant “P06” reported a technical difficulty, as he was unable to open a web link in the app. Regarding the app design, some participants found the mascot, graphics, and color scheme “visually appealing and beautiful” (P08) while others considered that older users may not connect with the design. These opinions were reflected in the attractiveness ratings (median 1, IQR –1 to 3).

### Considering and Preparing for Cardiac Rehabilitation

Both the videos and informational content helped users understand CR as a key component of their recovery from the cardiac event and validated the decision to attend CR or not. The representation of the pathway signposted out the way to CR step by step, offering a sense of security and direction. Participants “P05” and “P08” proposed to even extend the pathway and include tips for navigating the phase following a CR. For most participants, however, the app represented a guide for CR preparation. The note-taking feature enhanced this by nudging them to record relevant details. Participant “P01” described being called by the CR center and told his program would start in a few days. Overwhelmed, he used the app to gather necessary information and write a packing list:

*The app was really helpful. I got admitted so quickly. […] For me it was just: hurry up and pack! […] So I sat down, looked through the app, and it showed me everything I needed.*

[P01]

The link to the “rehabilitation compass,” an official website in Austria that informs about rehabilitation options near one’s location, supported some participants in selecting the most suitable option. This feature was especially relevant for participant “P03”, who decided not to participate in a CR program because the only rehabilitation center near his home did not offer CR services:

*The compass was very helpful for me because it showed where the CR centers are located. I saw there was a rehabilitation center near me, but it was not an option because it does not have a cardiovascular department.*

[P03]

### Enhancing Motivation to Return to Daily Activities

Some participants noted that after a cardiac event, one might either try to ignore it or overanalyze its causes. Therefore, they appreciated that the videos addressed uncertainties and fears after a cardiac intervention, showing how the individuals dealt with those challenges. For example, participant “P04” shared that the videos encouraged her to return to her gym training and to talk to relatives about her cardiac event:

*The video really got me thinking. That was when it finally sank in what actually happened to me. It was a real ‘aha’ moment. I had never really understood it before. […] It suddenly hit me that I almost died, and you have to process that. It was awful. […] I had a day or two where I did not want to be around anyone. It felt really strange. That is why it is so important to talk with people, you need to let it all out so you can handle it better.*

[P04]

Further, the HERO app motivated some participants to set goals for the phase after hospital discharge and for CR. For example, participant “P06” mentioned that using the app taught him “to start setting daily goals at home, to make sure I do something, to get moving. That was an interesting realization for me” (P06). The displayed pathway further motivated the participants by showing the way back to “normality” (P01) and fostering a sense of optimism that “everything will be okay, things are looking up” (P04). It also led to a self-reflection, as expressed by participant P05: “It made me realize how much I’ve already accomplished.*”*

### Supporting Sense-Making of the Cardiac Event

Overall, the video content was perceived as valuable for peer connection, helping participants navigate their rehabilitation journey beyond medical information alone. Some reported that the videos provided a sense of belonging and peer support in moving past the constant worrying about the cardiac event:

*[The videos] show that you’re not alone in what you’re going through, and I think that’s really, really important*

[P07]

*The videos showed me that other young and fit people had gone through the same thing. That really helped me break out of my cycle of overthinking*

[P05]

Some participants also shared that hearing about more severe cardiac diseases made them reconsider the severity of their own experience:

*Listening to others who went through worse made me think: Okay, my case wasn’t that bad after all.*

[P04]

While some participants could easily relate to the portrayed individuals, others found them less representative of their situation. In line with this finding, one of the most frequently mentioned ideas was to include more videos featuring different individuals, such as diverse age groups, genders, and cardiac conditions, allowing users to relate more closely to those experiences:

*The guy basically has my story, he had a heart attack, and probably has cholesterol problems, but he’s super skinny! […] I would have liked to see someone who has my vibe [laughs]. Someone overweight. […] It would be cool if, when I open the app, I could see someone who is in my demographic.*

[P06]

### Ensuring Timely and Location-Independent Access to the App

Most participants found the HERO app useful in their everyday lives (median 4.5, IQR 2-6) and generally enjoyed using it (median 5, IQR 4-6). All participants emphasized that earlier access to the app would have been even more beneficial; they stated that they would have liked to use it during their hospital stay to receive early information about follow-up care. Some participants had already actively searched for information on the web during their hospitalization or were familiar with the rehabilitation program from previous experiences. As a result, they reported that the app provided little new content for them:

*I started gathering information while I was still in the hospital […]. I had a heart attack. It came as a big surprise, given that I do a lot of hiking and cycling. I’m not even 60 yet, turning 60 in two weeks. […] So, I spent a lot of time looking into what I could do and what would happen next, even before I had the app. That’s why I already knew quite a bit.*

[P02]

The participants emphasized that having the app readily available on a smartphone was advantageous, as it allowed for location-independent access at any time. They used the app both at home and on the go. For example, participant “P01” described using the note-taking function while shopping for sportswear in preparation for his CR stay:

*You are out shopping, you have got your packing list written down, and you open the app like, “Oh, right, I need this, that, and that.” It is awesome. […] I do not have to walk around juggling a bunch of handwritten notes anymore, it is all in one place.*

[P01]

### Improving Self-Management Guidance and Addressing Information Gaps

Some recommendations in the HERO app were considered too vague, leading to dissatisfaction. For example, the information to ask insurance providers or general practitioners was perceived as not being helpful. Participants feared being stuck in phone queues or that health care professionals would not take their concerns seriously, particularly when they felt uncertain about managing their cardiac condition. Therefore, they requested more concrete information regarding lifestyle and coping strategies for the posthospital phase, with detailed tips on exercise, nutrition, and stress management. Participants suggested a feature for storing medication details, mentioning the possibility of side effects, and health care professionals’ contact details. Also, a direct download link for the CR referral form was suggested. Some wanted clearer insights on insurance and cost coverage. Several participants emphasized the importance of providing novel information in the app, offering details not readily available on rehabilitation centers’ websites. For example, they suggested including positive messages to reinforce the idea of turning the cardiac event into an opportunity to adopt a healthier lifestyle.

## Discussion

### HERO App as a Short-Term Tool to Bridge Patient Transitions

This exploratory study researched how the HERO app, an mHealth technology designed to guide patients from hospital discharge to CR, can support individuals during a critical care transition. The app was mainly perceived as a short-term tool to bridge the phase after hospital discharge, after which it can be set aside again. The participants emphasized the value of accessing the app early, ideally during their hospital stay, to receive timely information. They also valued the combination of evidence-based information about CR and experiential knowledge provided by peers to self-manage their patient pathways effectively. Patients felt supported in their decision-making about CR participation by receiving objective information about CR, which was presented as one option for follow-up care without pressuring them to participate. This is in line with the understanding of shared decision-making described by Elwyn et al [33], stating that no participation is also a valid option. We also identified areas for improvement, particularly in the personalization and concreteness of self-management support.

### Implications for Integrating mHealth Into Transitional Care Contexts

#### Overview

**Figure 2.**
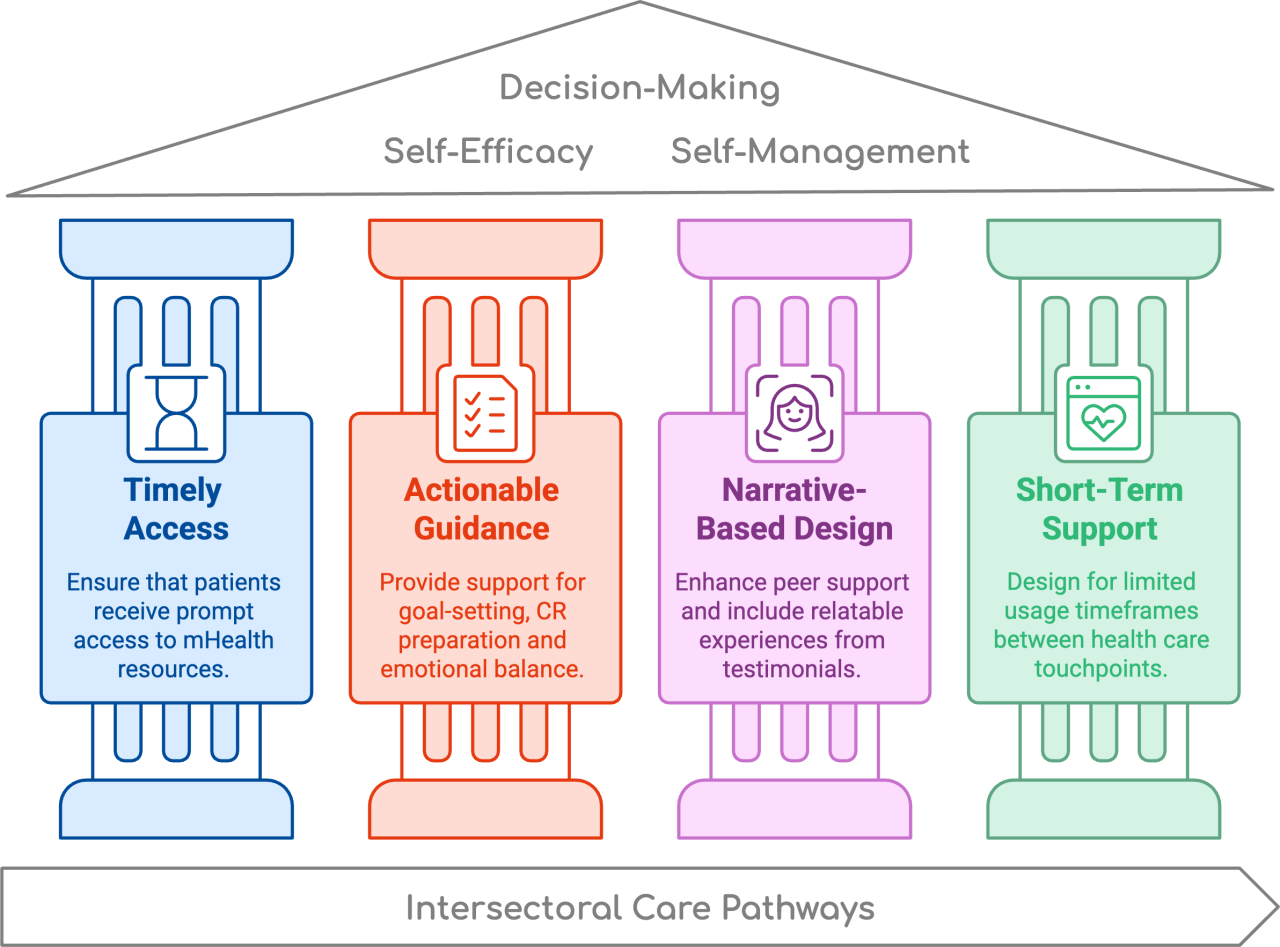
Four pillars of mHealth for cardiac care that can support patient decision-making, self-efficacy, and self-management during intersectoral care transitions. CR: cardiac rehabilitation; mHealth: mobile health.

Based on our findings, we derived four implications for designing and integrating mHealth technologies for patient support between 2 health care touchpoints: (1) Ensure timely access to mHealth support, (2) provide actionable guidance for self-management, (3) include peer narratives for emotional reassurance, and (4) design for short-term time frames. Figure 2 visualizes the implications as 4 pillars that support patient decision-making and can enhance their self-efficacy and self-management skills. The model could inform the development of patient-centered digital technologies by addressing their need for timely, actionable, and emotionally supportive guidance. Future research could apply this model to design, adapt, or evaluate mHealth interventions across different medical contexts where patients face similar challenges in managing care transitions.

#### Implication 1: Ensure Timely Access to mHealth Support

Our study highlights the importance of early access to information provided by mHealth, as participants expressed a need for immediate guidance after a cardiac event to support emotional processing, orientation, and the initiation of self-management while being hospitalized. Due to the recruitment process, we were only able to provide the app to patients after their discharge from the acute hospital. For some, this was too late, and they had already gathered information through other sources. To ensure timely access, we argue that mHealth technologies, such as the HERO app, should be integrated into patient pathways and discharge protocols. For example, a combination of automated referral technologies and mHealth support for patients may help to initiate patient pathways within the hospital setting [34]. Targeted implementation strategies based on a holistic context analysis provided by the NASSS framework, for example, could provide support here [25,35].

#### Implication 2: Provide Actionable Guidance for Self-Management

A key finding of this study was that participants expressed a strong need to actively manage their recovery and navigate their care pathway with a cardiac disease. To achieve this, having all information stored in 1 place was perceived as useful, highlighting the potential of mHealth in reducing patients’ burden of searching for and storing information from multiple sources [36,37]. Providing step-by-step guidance for organizing daily life and tailored information according to the pathway progress can support the navigation of health and administrative challenges. Our study showed that patients prefer concrete, actionable guidance over general recommendations; they request tips on physical activity, nutrition, stress management, and administrative steps for CR access. However, for patient safety, some types of advice, such as information on individual medication plans, should be personalized and provided by qualified health care professionals only. We also argue that, from a practical standpoint, the more information is incorporated into the app, the more it needs to be updated and quality-checked on an ongoing basis.

Nevertheless, the need for such content highlights a readiness to engage in self-management but also a reliance on (digital) tools to do so effectively. Participants emphasized that the app should display information in addition to what they receive from websites and health care professionals, indicating that they trust the app’s content and consider the displayed information as reliable. The HERO app offered a foundation for self-management by providing a visual CR pathway, a note-taking feature, and videos from former rehabilitation participants. These elements helped participants reflect on their condition, set goals, and regain a sense of agency and empowerment. According to the health action and process approach [38], a model for understanding health behavior change, forming intentions is based on risk perception and self-efficacy, factors that were activated by participants’ engagement with the app. For instance, several participants described the video content as eye-opening, helping them realize the seriousness of their condition and set small goals, such as organizing logistics for CR or integrating physical activity into their daily routines. In line with this, Schneider-Matyka et al [39] found evidence that information influences patients’ coping strategies, leading to less avoidance and more active engagement with their recovery.

#### Implication 3: Include Peer Narratives

The videos from testimonials emerged as a central feature supporting patients’ emotional balancing and coping with their cardiac disease. On the one hand, the videos fostered a sense of belonging, helping users feel less isolated after their diagnosis that is confirmed by previous research [40]. Furthermore, participants reported that the videos prompted self-reflection and emotional processing, helping to overcome uncertainties about physical activity and enter the CR program feeling better prepared. Active engagement with experiences helped participants stop overthinking the cardiac event. This is considered a critical step before returning to daily activities, which builds the basis for self-efficacy and long-term behavior changes [38,41].

On the other hand, we found that participants’ identification with the testimonials varied, and some participants felt unrepresented due to differences in age, body type, or cardiac history. Sillence et al [28] state that patients could reject information if it does not resonate with their own experiences or health conditions. Based on our findings, we partly agree, but we also want to highlight an additional facet of peer support. We found that contrasting different conditions can be helpful for some patients. The videos encouraged social comparison, allowing our participants to contextualize their condition. To enhance the potential of peer support, diversity in age, gender, and diagnosis should therefore be prioritized to ensure broader identification and inclusiveness [27,42].

#### Implication 4: Design for Short-Term Support

One interesting pattern we observed was that participants rated the app as useful and enjoyable; however, they expressed low intention to continue using it. The qualitative data help explain this disconnect. Many participants viewed the HERO app as a short-term tool to support the period between hospital discharge and the start of CR. Once CR began, they no longer felt the need to engage with the app. This supports the idea that mHealth tools for care transitions may not need long-term engagement to be effective but instead should focus on timely, targeted support. Unlike long-term mHealth interventions designed to sustain behavior change, mHealth used by patients in transition phases should include tailored content that can be quickly accessed with minimal onboarding and designed for limited usage time frames. Tools, such as the HERO app, aim to activate self-management and promote readiness rather than facilitate continuous tracking or habit formation. This distinction highlights the importance of tailoring mHealth technologies to short-term engagement, for example, by supporting brief patient interactions with tools for quick reference or note-taking [19].

### Limitations and Implications for Future Research

Our decision to close data collection after 8 participants was based on several considerations. First, we were able to recruit a sample, including variation regarding age, gender, years of living with cardiovascular disease, perspective on the patient pathway, and previous CR experience. Following Guest et al [43], who found that thematic saturation often occurs between 6 and 12 interviews in homogenous qualitative studies, we concluded that our 8 interviews were sufficient to capture the main themes. Recurrent themes appeared across the participants, indicating that our research questions had been comprehensively addressed. Nevertheless, participants’ experiences were shaped by the specific Austrian health care context, which may affect transferability to other systems. Second, data analysis was conducted in parallel with continued interviewing. This process allowed us to constantly assess the data richness and realize the point of data saturation when additional data would likely lead to redundancy rather than new findings. Third, recruitment in the acute hospital setting proved to be exceptionally challenging due to patient availability. Another significant barrier to participant enrollment was that the app was only available for Android devices, excluding iOS users from participation. Consequently, several interested individuals were unable to participate, which may have introduced a selection bias favoring Android users. Future iterations of the HERO app are planned to include iOS compatibility to ensure broader accessibility. Given these limitations, we assessed data saturation pragmatically, consciously balancing the data richness with recruitment constraints.

Furthermore, our recruitment approach may have resulted in selection bias regarding language skills, formal education levels, and personal interest in digital tools. Consequently, the perspectives of patient groups typically at risk of being underinformed, such as those with lower education levels or non-German-speaking backgrounds, may not be fully represented.

To ensure app uptake directly from a hospital bed, further refinements of the HERO app should improve accessibility, for example, by offering a more comprehensive onboarding process that explains key app features in greater detail, such as through a video walkthrough. This could improve ease of use and strengthen both initial engagement and perceived usefulness [20]. Due to budgetary and technical constraints, the current HERO app prototype does not include condition-specific content (eg, for patients recovering from cardiac surgery). Future iterations should incorporate more individualized information to reflect different patient experiences and care needs. Research shows that personalized content increases engagement with mHealth tools, and tailoring content to individual needs is a key factor in sustained use [20]. Adapting the HERO app for specific patient groups, such as postsurgery versus patients after a myocardial infarction, could therefore enhance both its relevance and long-term impact.

Following these improvements, a structured evaluation of the effects of the HERO app on pathway adherence, patient activation, and health literacy would be valuable. A randomized controlled trial with extended follow-up periods would be well-suited for this purpose. In addition, targeted implementation strategies could be developed to support the integration of mHealth tools into clinical routines [35]. Combining sociotechnical frameworks and user-centered design principles could be a promising approach [44]. Finally, future studies should examine the role of health care professionals in integrating mHealth into practice and in supporting patients in using such tools effectively [45].

To conclude, we would like to discuss the applicability of our 4-pillar model to other medical fields in which patient transitions between health care settings are relevant, such as orthopedics. While the challenges faced by patients may vary, such as the fear of mortality in patients with cardiovascular disease versus mobility concerns in patients with orthopedic conditions, both groups share insecurities about managing their new health conditions. Scott et al [46] highlighted that patients with orthopedic conditions could experience depression and anxiety, significantly impacting their overall recovery and quality of life. In this regard, the 4 pillars identified in our study—timely intervention, actionable guidance, relatable peer narratives, and short-term support—could also provide valuable support in developing targeted mHealth interventions for orthopedic conditions. However, it is crucial to identify target group-specific needs and contextual factors before applying our findings to other medical fields.

### Conclusion

This exploratory field study investigated how mHealth technologies, such as the HERO app, can support patients with cardiovascular disease in navigating care transitions from the acute hospital to CR. The app helped participants “break out of the cycle of overthinking” *(*P05) by providing short-term support, enabling self-management, and supporting decision-making and self-efficacy. User experiences suggested that the HERO app could support patient transitions, particularly in considering and preparing for CR, enhancing motivation to return to daily activities, and making sense of the cardiac event. Based on the findings, we propose four pillars of mHealth support for cardiac care transitions: (1) timely access to information, (2) actionable guidance for self-management, (3) peer narratives for emotional reassurance, and (4) design for short-term usability. This model may guide the future design and implementation of mHealth tools across various medical contexts where patients navigate complex care transitions.

## Supplementary material

10.2196/76089 Multimedia Appendix 1 HERO app video walkthrough.

10.2196/76089 Multimedia Appendix 2 Guide for semistructured interviews with study participants.

10.2196/76089 Multimedia Appendix 3 Category system for data analysis.

## Abbreviations

CR: cardiac rehabilitation
mHealth: mobile health
NASSS: Nonadoption, Abandonment, Scale-Up, Spread and Sustainability
UTAUT2: Unified Theory of Acceptance and Use of Technology 2
