# Supplementary material for: mHealth Support in Cardiac Care Pathways for Patient Self-Management During Transitions From Hospital to Rehabilitation: Exploratory Field Study
Source: JMIR Cardio. 2025 Aug 27;9:e76089. doi: 10.2196/76089 (PMC12384674; doi:10.2196/76089)
Supplement: Multimedia Appendix 2 [file cardio-v9-e76089-s002.docx]

**Multimedia Appendix 2: Guide for semi-structured interviews with study participants**

| Start audio recording |
| --- |

| Introduction | *Think back to the time you spent using the app. Please describe the most memorable moment you experienced with it.* |
| --- | --- |
| Follow-up questions | What did you look at in the app?  Where were you when you used the app?  Why did you open the app in this moment?  What helped you in that moment?  How did you feel after using the app? |
| Talking about the functions | *Which features of the app did you particularly like?*  *Which features of the app did you like less?* |
| Improvement suggestions | *If time, money and feasibility were not an issue, what would you change about the app?* |
| Closing | *Are there any other experiences with the app you would like to share that we have not talked about yet?* |

| End audio recording |
| --- |
