# Supplementary material for: mHealth Support in Cardiac Care Pathways for Patient Self-Management During Transitions From Hospital to Rehabilitation: Exploratory Field Study
Source: JMIR Cardio. 2025 Aug 27;9:e76089. doi: 10.2196/76089 (PMC12384674; doi:10.2196/76089)
Supplement: Multimedia Appendix 3 [file cardio-v9-e76089-s003.docx]

**Multimedia Appendix 3: Category system for data analysis**

Deductively defined categories and definitions, based on the research questions, previous findings on cardiac patient needs and user experience [8,32].

| Category name | Definition |
| --- | --- |
| System character | Usability and functionality of the HERO app, especially efficiency and satisfaction regarding access to content; perceived ease of use; design and aesthetics of the app; enjoyment; the perceived usefulness |
| Context | Environments and situations in which interaction with app happened or where it should happen along a patient pathway |
| Users’ internal state | Users’ predispositions, mood, and how the content and function influenced their need for orientation, need for motivation and need for emotional balance |
| Ideation | Suggestions on how the app’s content could be expanded and how its functions could be improved |
